# Jitter Elimination in Shape Recovery by using Adaptive Neural Network Filter

**DOI:** 10.3390/s19112566

**Published:** 2019-06-05

**Authors:** Sung-An Lee, Hoon-Seok Jang, Byung-Geun Lee

**Affiliations:** 1 School of Electrical Engineering and Computer Science, Gwangju Institute of Science and Technology, Gwangju 61005, Korea; ggamsi88@gist.ac.kr; 2 Three Dimensional Display Lab, Center for Imaging Media Research, Korea Institute of Science and Technology, Seoul 02792, Korea; jhs0053@kist.re.kr

**Keywords:** SFF, 3D cameras, 3D shape recovery, jitter noise, adaptive neural network filter

## Abstract

Three-dimensional (3D) cameras are expensive because they employ additional charged coupled device sensors and optical elements, e.g., lasers or complicated scanning mirror systems. One passive optical method, shape from focus (SFF), provides an efficient low cost solution for 3D cameras. However, mechanical vibration of the SFF imaging system causes jitter noise along the optical axis, which makes it difficult to obtain accurate shape information of objects. In traditional methods, this error cannot be removed and increases as the estimation of the shape recovery progresses. Therefore, the final 3D shape may be inaccurate. We introduce an accurate depth estimation method using an adaptive neural network (ANN) filter to remove the jitter noise effects. Jitter noise is modeled by both Gaussian distribution and non-Gaussian distribution. Then, focus curves are modeled by quadratic functions. The ANN filter is designed as an optimal estimator restoring the original position of each frame of the input image sequence in the modeled jitter noise, as a pre-processing step before the initial depth map is obtained. The proposed method was evaluated using image sequences of both synthetic and real objects. Experimental results demonstrate that it is reasonably efficient and that its accuracy is comparable with that of existing systems.

## 1. Introduction

Three-dimensional (3D) cameras with depth sensing capabilities are now widely used in many areas including research, education, security, entertainment, medicine, and the semiconductor industry. These cameras typically employ active optical methods, e.g., the projected texture stereo or triangulation methods and time of flight using a laser scanner, a charged coupled device (CCD) sensor, and projector. However, these methods are expensive. The cost can be reduced by applying passive optical methods. During the last decades, shape from focus (SFF), a passive optical method, has provided a solution that is cheaper and more efficient than other 3D shape recovery techniques, because of its simplicity and the accuracy of the system configuration. In this system configuration, there is no need for additional optical elements beyond the CCD camera. Hence, the techniques have been widely used in the consumer electronics field [1,2,3,4,5]. The goal of SFF is to detect the 3D depth of every point of an object from a camera lens using focus cues. An image sequence of the object is obtained from a fixed point of view, changing the distances between lens and object consecutively with a predefined small step. Then, SFF infers the 3D shape of the object from the image sequence.

The research on SFF techniques are mainly divided into three categories, focus measure (FM), approximation techniques, and optimization. FM uses the local sharpness measure of each pixel on an image. The initial depth map is obtained by finding the maximum responses of FM on every pixel. Many different FM operators have been invented, including sum of modified Laplacian (SML) [6], Tenenbaum (TEN), gray level variance (GLV) [7], mean method focus measure (FMM) [8], optical focus measure (FMO) [9], sum of wavelet coefficients (SWCs) [10], and eigenvalues-based (EB) [11]. However, methods that only use FM are not sufficient to reconstruct an accurate 3D shape due to the information loss between consecutive frames. Therefore, approximation techniques have been invented to refine the initial depth calculation by including various focus curve fitting methods, e.g., planar and curved approximations for finding the exact focused image surface [12]. On the one hand, optimization techniques have been applied using machine learning algorithms and optimization techniques, including neural network [13], dynamic programming [14], and fuzzy logic [15] for better accuracy and higher speed. However, these SFF techniques do not consider mechanical vibrations in the image acquisition process of the system. In the acquisition process, jitter noise occurs along the optical axis in every step. Because the error or flaw of the obtained two-dimensional (2D) images due to the jitter noise will be carried forward to the next stage, erroneous 3D shape recovery results are acquired. A conventional way to solve this problem is to apply a pre-processing low pass filter, e.g., a Gaussian filter, to the image sequence. However, this does not sufficiently reduce jitter noise effects. Therefore, the results are not very accurate [16]. In the literature, various filters can be used for removing the jitter noise [17,18,19]. Recent research has applied Kalman filter to remove the jitter noise in SFF. In previous studies, the jitter noise has been modeled by the Gaussian distribution or Levy with finite variance. However, noise statistics are generally unknown in the real world. A Kalman filter can only perform optimally when the noise statistics for the measurement and process are completely known. Therefore, conventional methods based on a Kalman filter are not efficient for the system under practical noise situations.

In this article, the jitter noise is modeled by both Gaussian distribution and non-Gaussian distribution. Then, the focus curves are modeled by quadratic functions. Finally, we propose to apply an adaptive neural network (ANN) filter to each image frame in the image sequences to remove the jitter noise. Because of their nonlinear characteristics, ANN filters can be used to approximate any arbitrary function. The neuron is trained to learn to cancel the jitter noise. The designed filter was tested using both synthetic and real objects. Our two contributions can be summarized as follows: (1) the proposed approach covers a more practical noise situation by modeling noise as Gaussian and non-Gaussian; and (2) the proposed filter gives a better result than applying an optimum Kalman filter, which introduce some inevitable phase distortion in practical noise situations. In practical systems, statistics of noise are usually unknown and the filters based on Kalman may approximate those parameters with loss of optimality. Therefore, the filters based on Kalman estimation errors may increase indefinitely, and may even cause filtering divergence. The proposed adaptive network can be used to adjust the filter parameters recursively based on the input data stream in a system whose parameters are unknown. The experimental results demonstrate that the proposed model is significantly more efficient while maintaining an accuracy comparable to the SOTA techniques.

## 2. Noise Modeling

**Figure 1:**
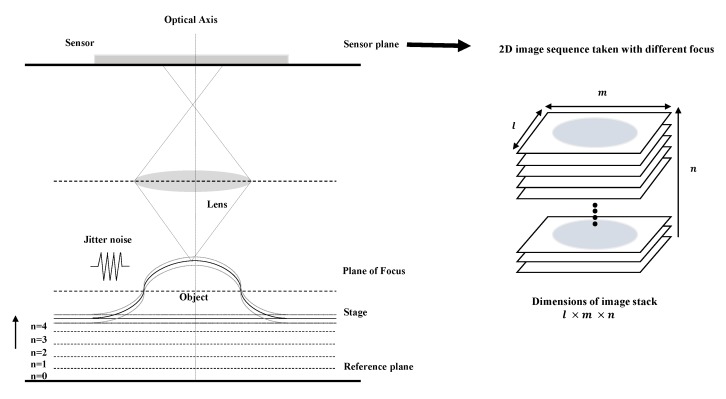
Principle of image sequence acquisition in SFF.

In SFF, a CCD camera is used to capture a sequence of images with different focus settings. The translational stage is moved along the optical axis from the reference plane in small steps. In each step, mechanical vibrations, called jitter noise, occur, as shown in Figure 1. Jitter noise is mainly divided into random jitter and deterministic jitter. Random jitter typically follows a Gaussian distribution and deterministic jitter has a known non-Gaussian distribution. If jitter has a Gaussian distribution, it is usually quantified using the standard deviation of this distribution. Often, jitter distribution is significantly non-Gaussian in practice. In this article, the jitter noise is modeled by using Gaussian and Lévy distribution. Lévy distribution is often used as non-Gaussian function. In this article, we model Gaussian and Lévy distribution in a manner similar to that of two prior studies [17,18]. The Gaussian probability density function (PDF) is completely described by $N(p,\sigma_{p})$. The mean *p* is the position of each image frame in the image sequence. For real objects, the standard deviation $\sigma_{p}$ is randomly selected among values to be <10 mm. The Lévy PDF varies only by two parameters, namely stability index α and scale parameter *c*, and is valid only for 0<α≤2 and c<0. The Fourier transform of Lévy PDF is defined as

(1) $$p\left(\Delta z\right)=\frac{1}{2\pi }\int exp\left(-ix\Delta z-{\left|cx\right|}^{\alpha }\right)dx$$

where δz is the difference between the position of image frame and that of corrupted by jitter noise. In our case, α and *c* are set to 1.3 and 10, respectively.

## 3. Focus Curve Modeling

In this section, the focus curves acquired by using the FM operator are modeled by quadratic functions to account for the effects of the jitter noise. The sharpness (or narrowness) of the focus curve plays an important role in the reliability of the depth estimation, because relatively flat peaks are highly sensitive to the localization of the maximum [20,21,22]. In our case, a quadratic polynomial model is enough to approximate the initial focus values. We define it as:

(2) $$F\left(z\right)=a_{2}z^{2}+a_{1}z+a_{0}$$

where *z* is the position of the image frames, F(z) is the FM values as functions of *z*, and $a_{0}$, $a_{1}$, and $a_{2}$ are coefficients of the quadratic function. To obtain these coefficients for every pixel, at least three arbitrary z positions and initial focus values are used. In this article, the initial focus values are obtained by applying SML. Finally, when *z* is changed to z+ζ, representing the noise, the modeled focus curve $F_{n}\left(z\right)$ is:

(3) $$F_{n}\left(z\right)=a_{2}{\left(z+\zeta \right)}^{2}+a_{1}\left(z+\zeta \right)+a_{0}=F\left(z\right)+a_{2}\left(\zeta^{2}+2z+\zeta \right)+a_{1}\zeta$$

In Equation (3), ζ is the jitter noise. In the next section, we filter it by using the proposed method to cancel the noise.

## 4. Proposed Method

**Figure 2:**
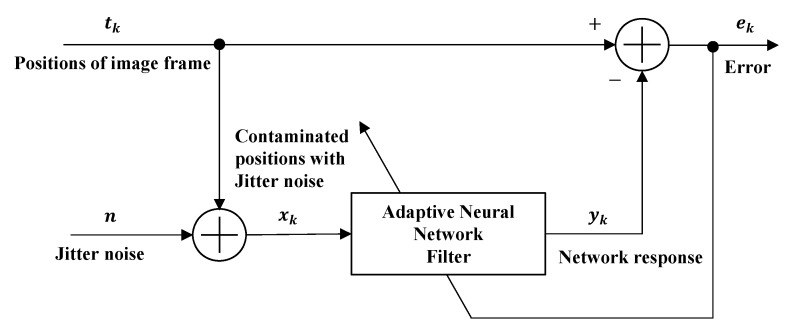
Noise cancelling of Adaptive Neural Network filter in SFF.

A common problem in signal processing is separating a signal from additive noise, also called interference cancelling [23]. Jitter noise due to mechanical vibration is one source of such noise. In the SFF system, the position of the image frame changes because of the jitter noise. Thus, the optimal position of each image frame should be obtained. In this section, we propose an ANN filter to remove the Gaussian and non-Gaussian noise as an optimal estimator in SFF system. An adaptive network can be used to model either a linear system whose parameters are unknown or a nonlinear system whose model is unknown. When knowledge of the noise statistics is unavailable a priori, it is still possible to develop a useful adaptive filter by using a recursive algorithm to adjust the filter parameters based on the input data stream. The network is adapted or trained by presenting it with successive input and desired vector pairs and then using the error signal to adapt the network to reduce the error consistently according to some specific learning rule. The ANN filter can be used to approximate any arbitrary function owing to its nonlinear characteristics. As shown in Figure 2, ANN can be used to cancel the signal noise. The process function is represented by the set of input and desired vector pairs used to train the network. The network filters the position corrupted by the jitter noise $x_{k}$ to produce an estimate $y_{k}$ of the actual position $t_{k}$. Then, it subtracts $y_{k}$ from the primary input $t_{k}$. The error signal $e_{k}$ becomes the estimate of the signal $t_{k}$. The proposed filter gives a better result compared with an optimum Kalman filter, which inevitably introduces phase distortion in practical noise situations. Here, the jitter noise is modeled by Gaussian noise and non-Gaussian and randomly examined to reflect a practical system environment.

**Figure 3:**
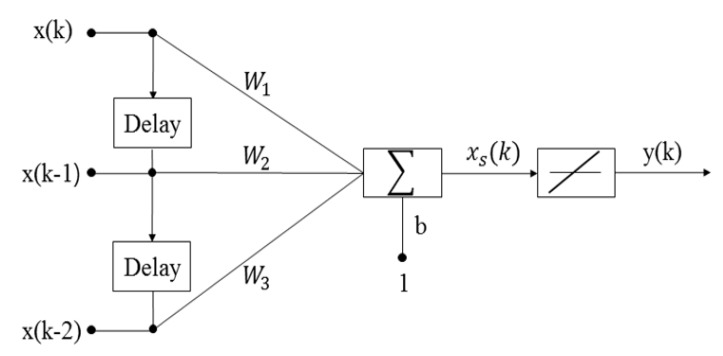
The design of proposed Adaptive Neural Network filter.

The proposed ANN filter is designed as shown in Figure 3. The filter has a tapped delay line with two delays, and we find the weights and bias that minimize the filter’s sum-squared error for recent input and corresponding target output. For the application of the filter to the SFF system, the input to the filter is defined by the position of each image frame in the 2D image sequence before filtering. The position of each image frame enters in order and passes through the two delays. The error function can be formed in many manners; however, the most common ways include the mean square error (MSE), least squares, weighted least squares, and instantaneous squared value. Strictly speaking, MSE is approximated by the other more practical methods because MSE is a theoretical value requiring an infinite amount of data. Therefore, to update the weights and bias as the iteration proceeds, MSE at the discrete instance *k* is defined as follows:

(4) $$MSE=\frac{1}{N}\sum_{N}^{k=1}{\left(e\left(k\right)\right)}^{2}=\frac{1}{N}\sum_{N}^{k=1}{\left(t\left(k\right)-y\left(k\right)\right)}^{2}$$

where *N* is the number of input to the filter, y(k) is the filtered output by the proposed method, t(k) is the corresponding target output, and e(k) is the error between the target output and the filter output. Here, N=3 because the filter has tapped the delay line with two delays. To obtain the optimal position of each image frame in the 2D image sequence, the weights and bias are adjusted using the least MSE algorithm based on an approximate steepest descent procedure as:

(5) $$W\left(k+1\right)=W\left(k\right)+2\alpha e\left(k\right)X^{T}\left(k\right)$$

(6) b(k+1)=b(k)+2αe(k)

where x(k) is the corrupted position at discrete instance *k*; X(k) is [x(k)x(k−1)x(k−2)]; W(k) is $\left[W_{1}\;W_{2}\;W_{3}\right]$; α is the learning rate; b(k) is the bias; and the superscript T indicates the transpose of the matrix. Here, the total number of iterations *I* of the adaptive neural network filter and α are set as 100 and 0.5, respectively. After acquiring filtered image sequence through iterations, a depth map is obtained by maximizing the focus measure acquired by using the previously modeled focus curve for each pixel in the image sequence.

## 5. Results and Discussion

### 5.1. Experimental Setup

**Figure 4:**
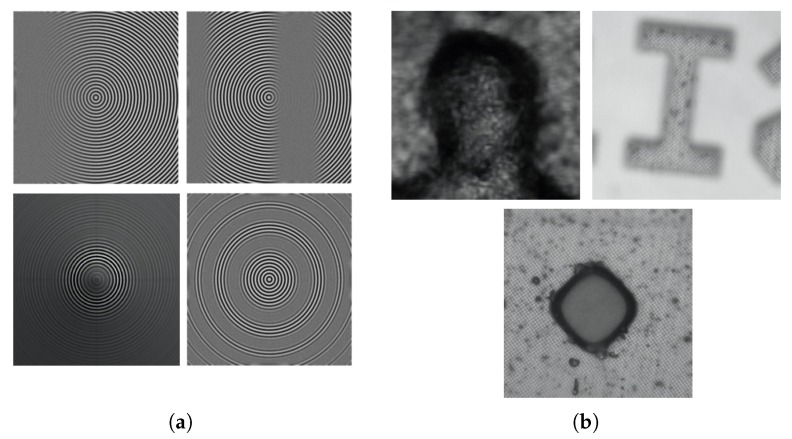
Sample frames of synthetic objects and real objects: (**a**) (left to right, top to bottom) plane object, sinusoidal object, cone object, and wave object; and (**b**) (left to right, top to bottom) Lincoln head on US penny, engraved letter-I, and LCD-TFT filter.

For the experiments, we used four synthetic objects, namely plane object, sinusoidal object, cone object, and wave object, created by a virtual program [24]. For each object, synthetic image sequence was generated by continuously varying focus settings. The cone object consisted of 97 frames of size 360×360 The other synthetic objects consisted of 80 frames of size 360×360 pixels. Real objects were obtained from a digital microscope control system, composed of a camera, a microscope, a motor controller, an optical axis motor, and a computer. The CCD camera was mounted on the microscope and the image sequences were acquired by controlling with movement the translational stage. Figure 4 shows sample frames of the synthetic objects and of the real objects used for the experiments: a LCD-TFT filter composed of 60 frames sized 300×300 pixels, a Lincoln head on US penny composed of 68 frames sized 300×300 pixels and an engraved letter I composed of 60 frame sized 300×300 pixels. In these experiments, the standard deviation of the measurement noise was assumed to be ten times the sampling step size of each image sequence, i.e., 254 mm, 1.059 μm, 6.191 μm and 1.529 μm for the synthetic objects LCD-TFT filter, US penny, and engraved letter I, respectively. The number of iterations of particle filtering and the proposed algorithm was 100. All experiments were carried out in MATLAB on a desktop computer with dual Cores Intel i7-6700k processors running at 4.0 GHz and with 32 GB RAM.

### 5.2. Quantitative Analysis

To evaluate the performance of the proposed algorithm compared to conventional methods, we used three quantitative metrics: root-mean-square-error (RMSE), correlation (Corr) and peak signal-to-noise ratio (PSNR). For synthetic objects, it was possible to calculate RMSE, correlation, and PSNR because their true depth maps are available. RMSE is an indicator of the discrepancy (error) between the true depth map and the estimated depth map. RMSE is the square root of MSE. It can be denoted as:

(7) $$MSE=\frac{1}{XY}\sum_{x=0}^{X-1}\sum_{y=0}^{Y-1}{\left|f\left(x,y\right)-g\left(x,y\right)\right|}^{2}$$

(8) $$RMSE=\sqrt{MSE}$$

where *X* and *Y* are the numbers of pixels in the horizontal and vertical directions of the input image, f(x,y) is the reference depth map, and g(x,y) is the restored depth map of the algorithm. Corr represents the similarity of two depth maps. It ranges from zero to one. A higher Corr value indicates that the restored depth map is more similar to the true depth map. It can be expressed as:

(9) $$r=\frac{\sum_{m}\sum_{n}\left(F_{mn}-\bar{F}\right)\left(G_{mn}-\bar{G}\right)}{\sqrt{\left(\sum_{m}\sum_{n}{\left(F_{mn}-\bar{F}\right)}^{2}\right)(\sum_{m}\sum_{n}{\left(G_{mn}-\bar{G}\right)}^{2}}}$$

where *F* and *G* are the reference depth map and restored depth map of the algorithm, with $\bar{F}$ and $\bar{G}$ their mean values. PSNR is the ratio between the maximum possible power of the original frames and the power of the corrupting noise. A higher PSNR indicates that the restored depth map is of higher quality. It can be defined using MSE:

(10) $$PSNR=20\log\left(MAX_{d}\right)-10\log\left(MSE\right)$$

where $\left(MAX_{d}\right)$ is the maximum possible pixel value of the restored depth map.

**Figure 5:**
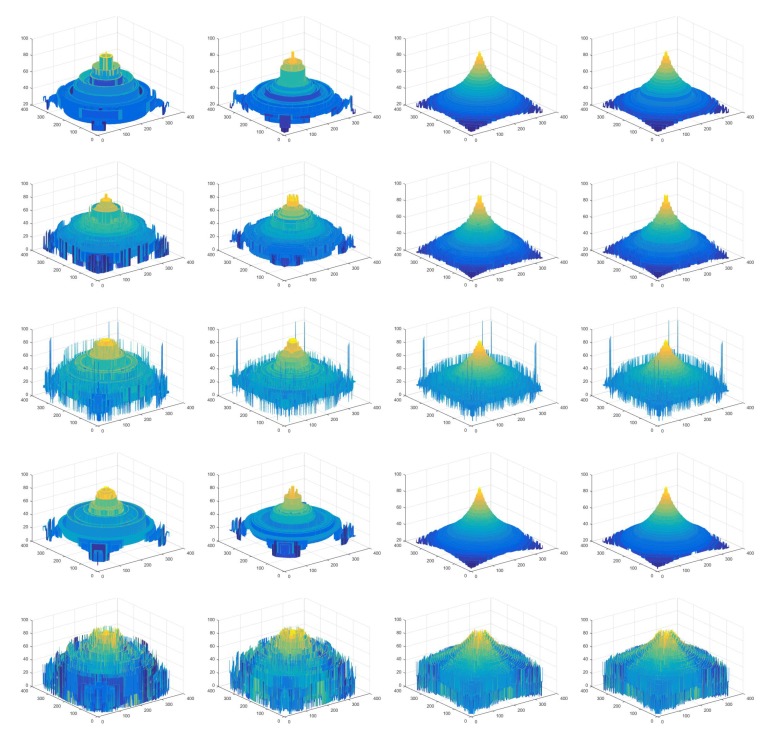
Restored 3D shape results for various focus measures using the cone object: first row, SML; second row, GLV; third row, TEN; fourth row, FMO; fifth row, FMM; first column, before filtering; second column, particle filtering; third column, MMSE filtering; and fourth column, the proposed filtering.

In the case of experiments for synthetic objects, it was possible to calculate RMSE, correlation and PSNR as their true depth maps are known, however it was impracticable to compute RMSE, correlation and PSNR metrics for synthetic objects as their true depth maps were not available. For comparisons and discussions of the proposed method, the depth map was estimated via five different FM operators. The other reason for using them was to show the effects of proposed filter on wide range of FMs. These FMs are the most commonly used for performance evaluation of 3D shape recovery. SML measures the sharpness of a pixel locally in the images by using the image Laplacian. GLV measures the variance of the gray-level of a pixel locally in the images. TEN is a gradient magnitude maximization method from the sum of the squares of each component of the two dimensional Sobel masks. FMM measures the focus values through the ratio of mean gray values among the neighboring pixels. FMO improves the performance of the 3D shape recovery in any noisy environment by using bipolar incoherent image processing. Before applying these FMs, the modeled jitter noise is added to each image sequence in the z-direction only. We experimented with jitter noise modeled in both Gaussian and non-Gaussian ways. For evaluating performance of the proposed ANN filter in the modeled of Gaussian noise, the particle filter, minimum mean square error (MMSE) filter and the proposed method were applied to remove these noise effects to recover the 3D shape and compared with the above three matrices. The particle filter is a conventional way to eliminate jitter noise and MMSE was used for comparing performance of jitter elimination [19]. Then, the depth map was obtained by choosing the sharpest pixels among the frames. Table 1 summarizes the performances in the four situations with respect to RMSE, Corr, and PSNR. The columns from left to right show the quantitative amounts of the restored depth maps of the cone object before filtering, after particle filtering, after MMSE filtering and after the proposed ANN filtering of the jitter noise. The FMO records the best result with respect to all three matrices. In the table, it is clear that our approach significantly improved all FM methods compared to the other filters. Figure 5 shows the restored 3D shapes of the cone object using the five FMs, with the last column showing the results of the proposed method. 3D shapes reconstructed through the before filtering have coarse surfaces due to jitter noise. Noisy focus values produce inaccurate depth values and relatively more spikes can be seen in the constructed depth maps of the objects. On the other hand, the proposed ANN filter reduced the effect of noisy focus measurement and provided more accurate depth maps. The ideal surface of synthetic cone object should be very smooth and the tip should be very sharp [14]. It can be observed that the 3D shape recovered by our ANN filer is smoother and finer than other filters. It demonstrates that the proposed ANN filter reduces the jitter noise more effectively in the 3D reconstruction than the particle filter and MMSE filter.

**Figure 6:**
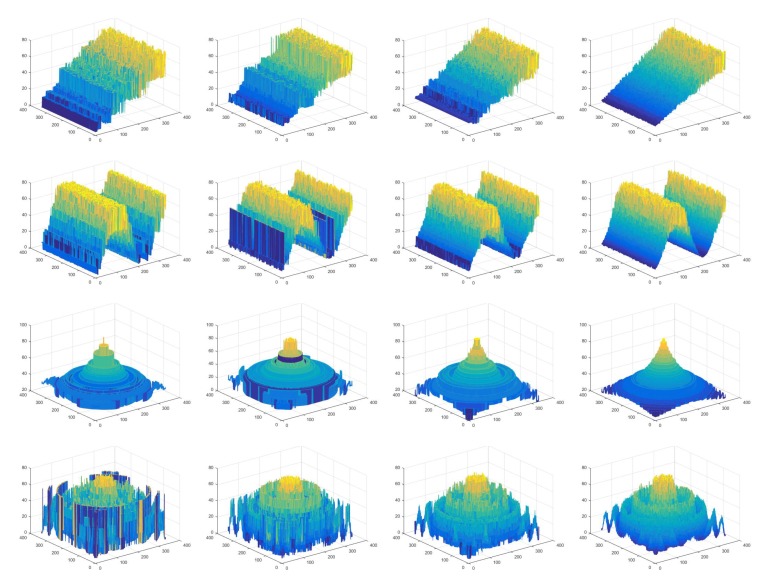
Restored 3D shape results for the synthetic objects using non-Gaussian filtering: first row, plane object; second row, sinusoidal object; third row, cone object; fourth row, wave object; first column, non-filtering; second column, Kalman filtering; third column, modified Kalman filtering; and fourth column, the proposed filtering.

To evaluate the performance of the proposed ANN filter in the modeled of non-Gaussian noise, Kalman filter, modified Kalman filter and the proposed filter were applied to remove these noise effects in the process of recovering the 3D shape and compared with the above two matrices. The Kalman filter and the modified Kalman filter are the most recently developed methods for jitter elimination on SFF system [17,18]. Table 2 shows the performances in the four situations with respect to RMSE and Corr. The columns from left to right show the quantitative amounts of the restored depth maps of the cone object before filtering, after Kalman filtering, after modified Kalman filtering, and after the proposed ANN filtering of the jitter noise. After these filters were applied to input images, FMO was used for depth estimation because it ensures reliable performance among the five FMs. In the table, it is clear that our approach significantly achieved the best performance for all synthetic objects. In the case of sinusoidal and cone object, the Kalman filter did not improve the RMSE and Corr compared to before filtering because it did not reflect the mechanical vibrations generated in real system environments with non-Gaussian noise. Figure 6 shows restored 3D shape results for the synthetic objects using the three non-Gaussian filters, with the last column showing the results of the proposed method. The sinusoidal and cone shape reconstructed by the Kalman filtering have the most coarse surface compared to others. It demonstrates that our method reduced the jitter noise more effectively in the 3D reconstruction than the Kalman filter and modified Kalman filter. It can be observed that the 3D shape recovered by our ANN filer is smoother than the other filters and the tips are also more shaply than others. The figures and tables clearly prove that our approach for filtering the jitter noise improved the 3D shape reconstruction, demonstrating the effectiveness of the proposed filer.

### 5.3. Qualitative Analysis

**Figure 7:**
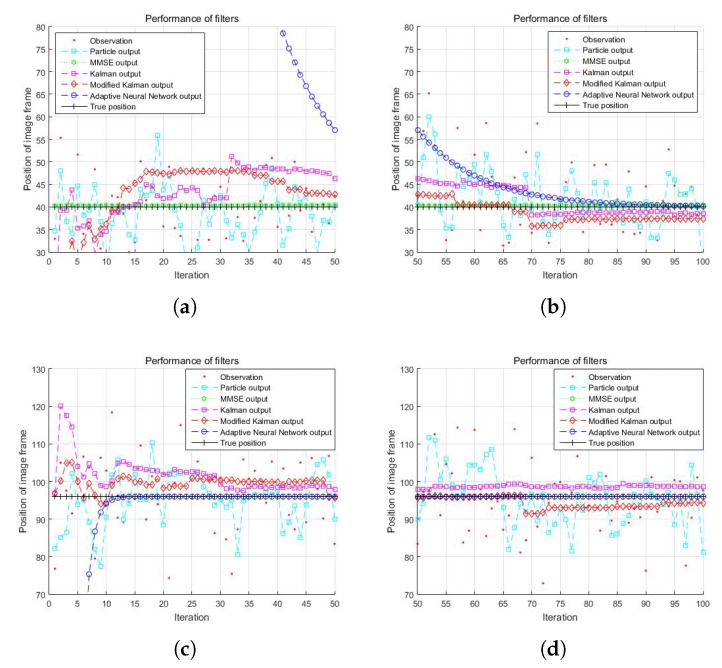
Performance of artificial neural network filter of two different image frames of the cone object: (**a**) 40th frame with Iterations 0–50; (**b**) 40th frame with Iterations 50–100; (**c**) 96th frame with Iterations 0–50; and (**d**) 96th frame with Iterations 50–100.

The total number of iterations *I* of the ANN filter was 100. Figure 7 shows the performance of the proposed method for the 40th and 96th frames of the cone object at different learning iterations. In the first column, when the number of total iterations was 0–50, the network produced inaccurate results (Figure 7a), but it converged quickly as the iteration increased. In the second column, when total iterations were 50–100, the network converged (Figure 7b). It is clear that the network requires sufficient iterations to converge to the optimal depth frame from the observation corrupted by jitter noise. When the amount of noise was small, even ten iterations were sufficient, as shown in Figure 7c,d. However, the amount of noise and the statistics thereof are not known in many practical situations. Thus, the number of iterations is decided empirically. There is a trade-off problem between the total number of iterations and time complexity. The number of iterations should be increased enough to converge; however, it causes time complexity. In our experiments, at least 100 iterations were required to ensure depth convergence for all pixels.

**Figure 8:**
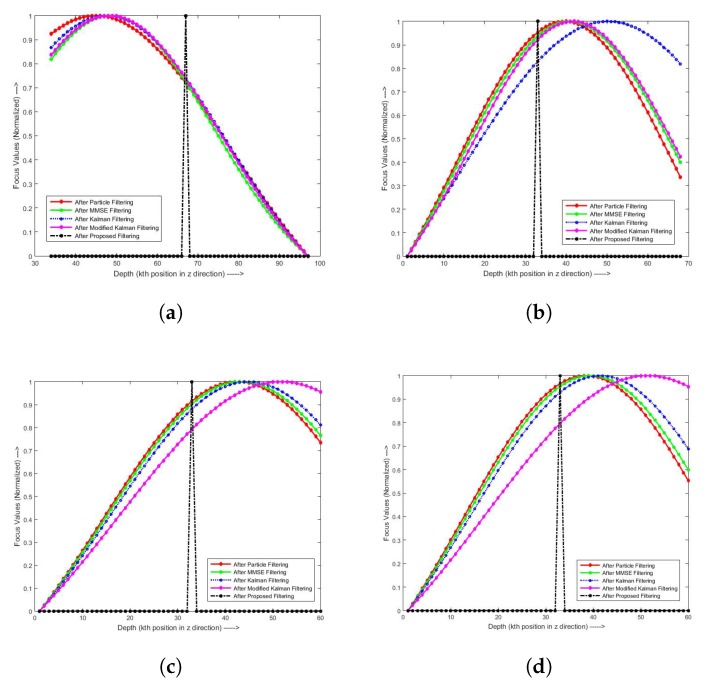
Focus curve approximation by quadratic function for the cone object, US penny, engraved letter I and LCD-TFT filter with different points: (**a**) cone object point (110,110); (**b**) US penny point (120,120); (**c**) engraved letter I point (130,130); and (**d**) LCD-TFT filter point (140,140).

Figure 8 shows the focus curves fitted to Gaussian model before and after Gaussian and non-Gaussian filters, for object points (100,100), (120,120), (130,130), and (140,140) on the cone object, US penny, engraved letter I, and LCD-TFT filter from each image sequence. The focus curves and the peak value of the focus curves were normalized to one for better visualization. In these figures, it is clear that the focus curves after the proposed filtering achieve sharper and narrower responses than the others. The literature shows that sharper and narrower focus curves generally produce better 3D retrieval [20,21,22].

**Figure 9:**
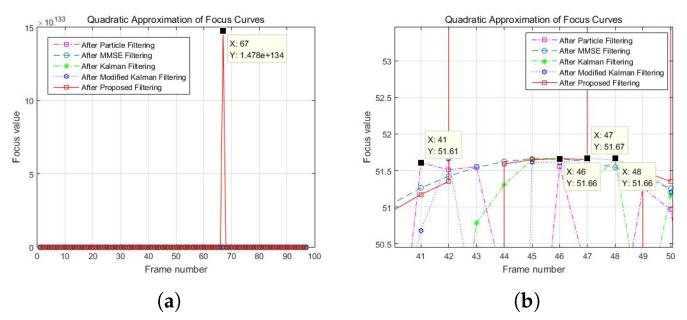
Comparison of the peak focus values for cone object: (**a**) cone object point (110,110); and (**b**) enlarged plot of (**a**).

Figure 9 shows the peak responses of the focus curves before filtering and using particle filtering and the proposed filtering. In our experiments, the peak of the focus value for the proposed filtering was at least $10^{100}$ times larger than the other methods for all object points.

**Figure 10:**
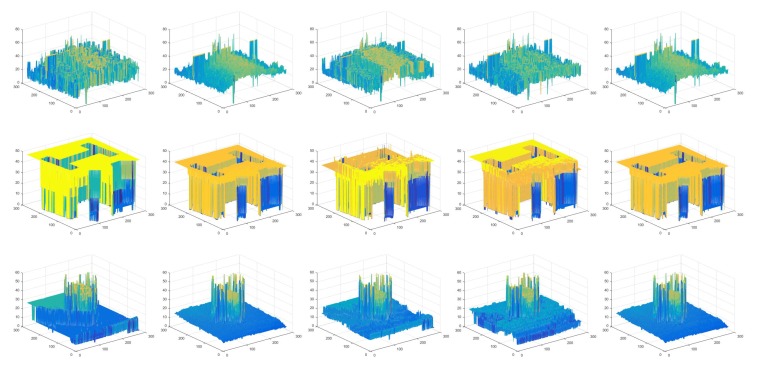
Restored 3D shape results for various objects using five kinds of filtering: first row, US penny; second row, engraved letter I; third row, LCD-TFT filter; first column, particle filtering; second column, MMSE filtering; third column, Kalman filtering; fourth column, modified Kalman filtering; and fifth column, the proposed filtering.

Figure 10 provides the 3D shape recovery results of three real objects by using five jitter elimination filters. The last column shows the results of our approach. It can be observed that the 3D shape recovered by our ANN filer is smoother and finer than other filters. It demonstrates that filtering the jitter noise using the proposed method improves the quality of the 3D shape recovery over using the existing approaches.

## 6. Conclusions

In the SFF system, mechanical vibration of the imaging system causes jitter noise along the optical axis and makes it difficult to estimate the shapes of objects accurately. We propose an adaptive neural network (ANN) for removal of jitter noise to improve the accuracy of the 3D shape retrieval techniques. We model the jitter noise by using Gaussian and non-Guassian distributions and focus curves as quadratic functions, respectively. Then, the ANN filter is designed and applied to remove this noise from the focus curves for all pixels. The experiments were conducted on four synthetic objects and three real objects, using five FMs, ensuring the robustness and effectiveness of our filter. To compare performance, the proposed algorithm was tested under two different noise situations with four different jitter elimination methods. Through experimental results, it is shown that applying the proposed method to previous SFF techniques improves the accuracy of 3D shape reconstruction; it works as a pre-process filter to remove the jitter noise. In addition, it improves the 3D shape reconstruction with better performance than the SOTA methods on the modeled Gaussian noise and the Lévy noise.

## Figures and Tables

**Table 1:** Performance comparison for various focus measures using the cone object.

Method	Before Filtering	After Particle Filtering	After MMSE Filtering	After ANN Filtering
	RMSE Corr PSNR	RMSE Corr PSNR	RMSE Corr PSNR	RMSE Corr PSNR
SML	11.6030 0.7804 18.2631	10.6967 0.7718 18.8776	8.2888 0.9395 21.2155	8.2117 0.9507 21.3567
GLV	11.2157 0.8231 18.7389	8.3104 0.8846 21.0701	7.6362 0.9548 21.8050	7.4629 0.9560 22.0963
TEN	11.1891 0.7099 18.7595	9.6286 0.8197 20.0642	9.0156 0.9056 20.9155	8.5519 0.9269 21.0041
FMO	12.5904 0.7567 17.3689	10.7549 0.7390 8.9224	7.9853 0.9106 21.5612	7.8107 0.9582 21.7007
FMM	19.1304 0.6671 14.1010	17.0982 0.6235 15.0764	16.5184 0.6911 15.8995	14.1171 0.7196 16.6505

**Table 2:** Performance comparison for Various SFF methods using various synthetic objects.

Object	Before Filtering	After Kalman Filtering	After Modified Kalman Filtering	After ANN Filtering
	RMSE (Corr)	RMSE (Corr)	RMSE (Corr)	RMSE (Corr)
Plane	4.589 (0.970)	4.196 (0.972)	4.073 (0.974)	3.805 (0.979)
Sinusoidal	4.649 (0.978)	4.630 (0.977)	4.505 (0.978)	3.782 (0.985)
Cone	8.548 (0.957)	8.567 (0.967)	8.503 (0.970)	8.462 (0.971)
Wave	2.824 (0.979)	2.581 (0.981)	2.366 (0.984)	2.004 (0.989)
